# The Comparative Analysis of the Ruminal Bacterial Population in Reindeer (*Rangifer tarandus* L.) from the Russian Arctic Zone: Regional and Seasonal Effects

**DOI:** 10.3390/ani11030911

**Published:** 2021-03-22

**Authors:** Larisa A. Ilina, Valentina A. Filippova, Evgeni A. Brazhnik, Andrey V. Dubrovin, Elena A. Yildirim, Timur P. Dunyashev, Georgiy Y. Laptev, Natalia I. Novikova, Dmitriy V. Sobolev, Aleksandr A. Yuzhakov, Kasim A. Laishev

**Affiliations:** 1 BIOTROF + Ltd., 8 Malinovskaya St, Liter A, 7-N, Pushkin, 196602 St. Petersburg, Russia; filippova@biotrof.ru (V.A.F.); bea@biotrof.ru (E.A.B.); dubrowin.a.v@yandex.ru (A.V.D.); deniz@biotrof.ru (E.A.Y.); timur@biotrof.ru (T.P.D.); georg-laptev@rambler.ru (G.Y.L.); natalia-iv-nov@rambler.ru (N.I.N.); sdv@biotrof.ru (D.V.S.); 2 Department of Animal Husbandry and Environmental Management of the Arctic, Federal Research Center of Russian Academy Sciences, 7, Sh. Podbel’skogo, Pushkin, 196608 St. Petersburg, Russia; alyuzhakov@mail.ru (A.A.Y.); layshev@mail.ru (K.A.L.)

**Keywords:** reindeer, rumen, microbiome, NGS, arctic zone

## Abstract

**Simple Summary:**

The reindeer (*Rangifer tarandus*) is a unique ruminant that lives in arctic areas characterized by severe living conditions. Low temperatures and a scarce diet containing a high proportion of hard-to-digest components have contributed to the development of several adaptations that allow reindeer to have a successful existence in the Far North region. These adaptations include the microbiome of the rumen—a digestive organ in ruminants that is responsible for crude fiber digestion through the enzymatic activity of microorganisms. In this study, research was conducted on the ruminal microbiome of reindeer of the Nenets breed living in various climatic zones of the Russian Arctic (in the Yamalo-Nenetski Autonomous District and Nenetski Autonomous District. The impacts of the habitat, season of the year, sex, and age factors on the rumen microbiome were investigated. As a result, it was found that significant differences in the reindeer ruminal microbiome composition are associated with the region of habitat and change of seasons that the reindeer are exposed to. The distinctions mainly come down to different ratios of bacteria involved in the metabolism of volatile fatty acids and cellulose decomposition in the rumen, which is apparently a reflection of the different plant components in the diet in different regions and seasons.

**Abstract:**

The reindeer (*Rangifer tarandus* L.) is a unique animal inhabitant of arctic regions. Low ambient temperatures and scant diets (primarily, lichens) have resulted in different evolutional adaptations, including the composition of the ruminal microbiota. In the study presented here, the effects of seasonal and regional aspects of the composition of the ruminal microbiota in reindeer (Nenets breed, 38 animals) were studied (wooded tundra from the Yamalo-Nenetski Autonomous District (YNAD) vs. from the Nenetski Autonomous District (NAD)). The ruminal content of calves (*n* = 12) and adult animals (*n* = 26, 15 males and 11 females) was sampled in the summer (*n* = 16) and winter seasons (*n* = 22). The composition of the ruminal microbial population was determined by the V3–V4 16S rRNA gene region sequencing. It was found that the population was dominated by Bacteroidetes and Firmicutes phyla, followed by *Spirochaetes* and *Verrucomicrobia*. An analysis of the community using non-metric multidimensional scaling and Bray–Curtis similarity metrics provided evidence that the most influential factors affecting the composition of ruminal microbiota are the region (*p* = 0.001) and season (*p* = 0.001); heat map analysis revealed several communities that are strongly affected by these two factors. In the summer season, the following communities were significantly larger compared to in the winter season: *Coriobactriaceae*, *Erysipelothrihaceae*, and *Mycoplasmataceae*. The following communities were significantly larger in the winter season compared to in summer: *Paraprevotellaceae*, *Butyrivibrio* spp., *Succiniclasticum* spp., *Coprococcus* spp., *Ruminococcus* spp., and *Pseudobutyrivibrio* spp. In NAD (tundra), the following communities were significantly larger in comparison to YNAD (wooded tundra): *Verrucomicrobia* (Verruco-5), *Anaerolinaceae*, PeHg47 *Planctomycetes*, cellulolytic *Lachnospiraceae*, and *Succiniclasticum* spp. The following bacterial groups were significantly larger in YNAD in comparison to NAD: cellulolytic *Ruminococaceae*, *Dehalobacteriaceae*, *Veillionelaceae*, and *Oscilospira* spp. The significant differences in the ruminal microbial population were primarily related to the ingredients of diets, affected by region and season. The summer-related increases in the communities of certain pathogens (*Mycoplasmataceae*, *Fusobacterium* spp., *Porphyromonas endodentalis*) were found. Regional differences were primarily related to the ratio of the species involved in ruminal cellulose degradation and ruminal fatty acids metabolism; these differences reflect the regional dissimilarities in botanical diet ingredients.

## 1. Introduction

The reindeer (*Rangifer tarandus* L.) is a unique ruminant that inhabits climatically severe arctic regions. The population of reindeer in Russia is ~3 million, including ~1 million wild animals [1]. Among the four breeds inhabiting the Russian Arctic zone (Nenets, Chukot, Even, and Evenki), the former is the most abundant. The reindeer is a vitally important animal for human populations of arctic zones, as it is a source of food, skins, fat, and antler-derived products [2,3].

The geographic isolation of reindeer from other Cervidae ruminants, in combination with the specific environmental conditions present, has resulted in certain morphologic and functional adaptations in the digestive system [4,5,6], including the specific microbial population of the rumen. The rumen is a digestive organ in ruminants where the major degradation of complex polysaccharides from botanical diet ingredients occurs through enzymatic systems of the symbiotic microbial population [7,8,9]. The ruminal microbial community includes prokaryotes (bacteria and archaea), protozoa, and fungi. The microbial communities in different ruminants are presently the subject of significant study due to the development of new concepts related to the roles of the entire microbial population and individual communities within it in the host animals [10,11,12,13,14,15]. The composition of the ruminal microbiota can be affected by different biotic and/or abiotic factors. Biotic factors include the diet composition, feed additives, genetics, and physiological status of the host organism (age, health). Abiotic factors include the season, region, and feeding schedule.

Interest in the symbiotic ruminal microbiota in reindeer is also related to the adaptations of this species to the harsh Arctic environment, including their ability to effectively utilize scarce botanical feed sources from the tundra, wooded tundra, and northern taiga zones [16,17,18].The Russian Subarctic tundra zone stretches from the Kola to Chukot peninsulae and features long (8–9 months), severe winters and short, cool summers (the annual period with an ambient temperature above zero is ~100 days). The flora is dominated by the lichens and mosses, while herbaceous and scrub plants are relatively scarce. The wooded tundra zone (the annual period with an ambient temperature above zero is ~120–130 days) includes a combination of the plant elements of tundra and woodland areas, including specific “open forests”; it is the wintering place for the majority of the tundra populations of reindeer [2].

The lichens that account for a large part of the vegetable feed resource of Arctic animals are known to synthesize a wide range of secondary metabolites, such as atranorin, and protocetraric and fumaroprocetraric acids [19], as well as usnic acid, which has antimicrobial and antifungal properties and is toxic to human and animals [20]. The scarcity of Arctic feed resources results in high consumption of different lichen species containing high concentrations of usnic acid by animals, even in the summer season. In the winter season, the percentage of these species in the diets of reindeer can increase to 70% [21,22]. These factors have contributed to the uniqueness of the ruminal microbial ecosystem in reindeer. Bacterial strains resistant to the toxic effects of usnic acid have been found in reindeer rumen [23]. It was also found that the ruminal microbial population can promptly neutralize toxic effects [24].

In addition to usnic acid, several factors evidently affect the composition of the ruminal microbiota in reindeer, including the variety of fiber sources, interactions between microbial communities, the diversity of cellulolytic enzymatic systems, physiological status of animals, and different ecological aspects [25,26].

The ecology of the place of inhabitation can affect the composition of the ruminal microbiota in different ruminant species [27]. For example, significant differences in ruminal microbial populations have been found in two geographically isolated subspecies of Norwegian reindeer: the Eurasian tundra reindeer (*R. tarandus tarandus*) inhabiting mainland Norway and the Svalbard reindeer (*R. tarandus platyrhynchus*) from the Spitzbergen Arctic archipelago between Norway and the Northern Pole. The total community of cellulolytic species in Svalbard reindeer (consuming diets with a high concentration of lignin for a winter season of 8–10 months) was found to be 6–14-fold larger compared to that of mainland tundra reindeer; however, the sets of microbial species related to the fermentation of vegetable diet ingredients were similar in these two reindeer subspecies and included the following species: *Peptostreptococcus anaerobius*, *Lachnospira multiparus*, *Butyrivibrio fibrisolvens*, *Eubacterium ruminantium*, *Selenomonas ruminantium*, *Fibrobacter succinogenes*, *Eubacterium pyruvovorans*, and *Fusocillus* spp. [28].

Investigation of the ruminal microbiota in different ruminant species revealed certain common influential factors affecting the composition: genotype [29,30] and age of the host [31], habitat area [28], season [21], diet and feeding schedule [32,33,34], health status [35,36], antibiotics application [35], and stress [37].

Despite the adaptability to life in the Arctic, reindeer are susceptible to various infectious and parasitic diseases, especially in the summer [38]. During this period, when the air temperature rises, which is especially important in connection with the process of global warming, animals expose to stress and often contract a disease [39]. It happens also due to the weakening of the animal’s body due to insufficient diet in the winter–spring period, which leads to a decrease in the protective functions. The reindeer husbandry industry suffers great losses due to the mortality from various diseases.

The ruminal microbiota in reindeer from Russian Arctic zones has not been comprehensively studied. The present study is the first comparative investigation of the ruminal bacterial population in reindeer from two different climatic zones of the Russian Arctic region: wooded tundra from the Yamalo-Nenetski Autonomous District (YNAD) vs. tundra from the Nenetski Autonomous District (NAD). The study also aimed to identify the basic factors affecting the ruminal microbial population, and the effects of certain internal and external factors (age, sex, season, diet) were assessed.

## 2. Materials and Methods

### 2.1. Sampling of Ruminal Content

**Figure 1:**
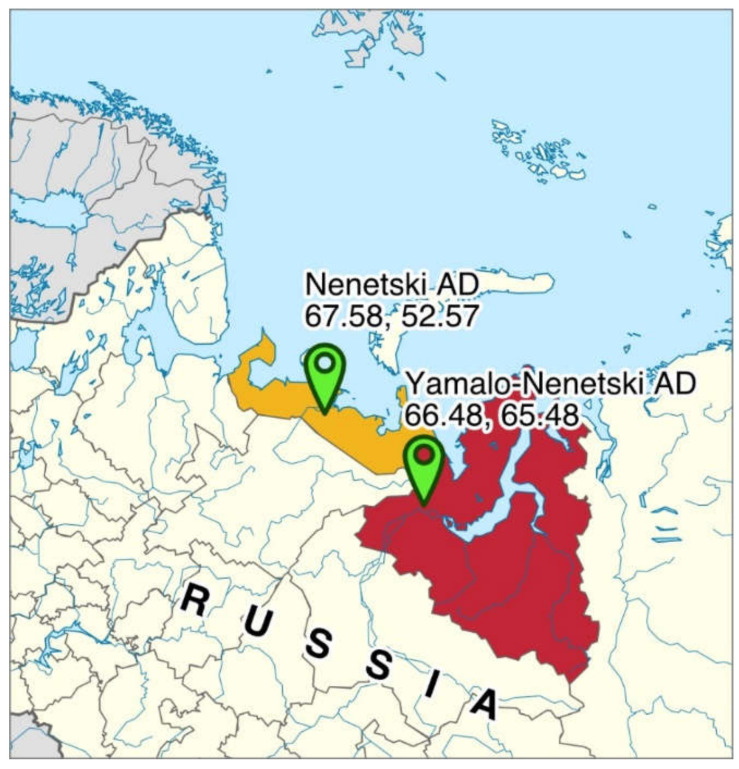
The locations from which the ruminal contents were collected from reindeer in the Yamalo-Nenetski Autonomous District (red) and the Nenetski Autonomous District (yellow).

The ruminal content of calves (4–8 months of age, *n* = 12) and adult (2–8 years of age, *n* = 26, 15 males and 11 females) reindeer (Nenets breed, total 38 animals) in the summer–autumn (*n* = 16) and winter–spring seasons (*n* = 22) of 2017–2018 was collected. This included 18 animals from the YNAD (settlement Harp, wooded tundra), including 6 samples collected in summer and 12 in winter, and 20 from the NAD (settlement Nelmin-Nos, tundra), including 10 samples collected in summer and 10 in the winter season (Figure 1, Supplementary Table S1).

The samples were collected using the aseptic flexible PVC tube in accordance with the principles of humanity, guidelines of the Declaration of Helsinki, and the national ethical rules for experiments on animals. Samples of chyme (30 mL) were taken from the upper part of the ventral rumen sac. The freshly collected samples were frozen and stored at −20 °C for subsequent isolation of the total DNA.

### 2.2. DNA Isolation and Sequencing

The total DNA content in the samples was isolated using the Genomic DNA Purification Kit (Fermentas, Inc., Vilnus, Lithuania) according to the producer’s manual. The isolated DNA was quantified with a Qubit^®^ 2.0 fluorimeter (Life Technologies, Carlsbad, CA, USA) and stored at −20 °C.

The composition of the ruminal microbial population was determined by next-generation sequencing (NGS) with the MiSeq system (Illumina, San Diego, CA, USA) in the V3–V4 region of the *16SrRNA* gene using the upstream primer 341F: 5′ TCGTCGGCAGCGTCAGATGTGTATAAGAGACAGCCTACGGGNGGCWGCAG, the downstream primer 805R: 5′ GTCTCGTGGGCTCGGAGATGTGTATAAGAGA CAGGACTACHVGGGTATCTAATCC, the Nextera^®^ XT Index Kit reagent kit (Illimina, San Diego, CA, USA) for preparation prior to sequencing, Agencourt AMPure XP for the purification of PCR products, and the MiSeq^®^ Reagent Kit v2 (500 cycle) for sequencing [40].

The processing of the reads obtained (including overlapping, filtration by sequence quality (Q30), trimming of the primers) was performed using Bioinformatic Software Tools (Illumina, San Diego, CA, USA). The quality control and analysis of the data were performed using QIIME2 ver.2019.10 (https://docs.qiime2.org, accessed on 12 August 2020) software [41]. After the import of the sequences into QIIME2 format, the paired read lines were aligned. Next, the sequence quality was filtered with default settings. Noise filtering using the Deblur tool was performed; the length of the sequences was left at a maximum of 250 bp. To construct phylogeny de novo, we applied alignment using MAFFT. For taxonomy assignment we used Greengenes reference database ver.13.5 99% (https://greengenes.secondgenome.com/?prefix=downloads/greengenes_database/gg_13_5/, accessed on 12 August 2020).

### 2.3. Statistical Analysis

The alpha-biodiversity indices for the comparison of the samples (including Shannon’s and Chao1) and prospective operational taxonomic units (OTUs) were analyzed using QIIME2 ver. 2019.10 (https://docs.qiime2.org, accessed on 12 August 2020) software [41]. The comparison of the communities was performed using non-metric multidimensional scaling (NMDS) and Bray–Curtis similarity metrics from the Vegan package for R [42].

The common and unique bacterial species in the rumens of different animals were calculated and visualized by the Venn Diagram package in R software [43,44]. To decrease the number of rare taxa, the analysis of the network of co-occurrence involved only taxa with an abundance of over 0.01% that were present in at least 50% of all samples (3 out of 6) from each animal.

To analyze the interaction of factors (season, age, region, sex), we performed ANOVA analysis. To exclude the type I error and to give statistical power to the model, the Tukey test correction was applied (https://www.rdocumentation.org/packages/stats/versions/3.6.1/topics/TukeyHSD, accessed on 12 August 2020).

The effects of different studied factors were assessed using adjusted *p*-values according to the method presented by Benjamini and Hochberg [45]. A heat map was built with the Pheatmap ver. 1.0.12 package for R (https://cran.r-project.org/web/packages/pheatmap/pheatmap.pdf, accessed on 12 August 2020). The data matrix was centered and scaled with subsequent clusterization according to Ward’s method [46] on the basis of the squared Euclidean distance matrix.

### 2.4. Accession Numbers

The *16S rRNA* gene sequences were deposited in the National Center for Biotechnology Information (NCBI) Sequence Read Archive (SRA) under BioProjects with the accession number PRJNA576999.

## 3. Results

### 3.1. Analysis of the Ruminal Microbial Population Biodiversity in Reindeer

The sequencing of the *16S rRNA* gene in the collected samples resulted in 1,058,032 sequences of acceptable quality, an average of 25,687 reads per sample. The sequence count varied from 5753 to 113,156 per sample, and the number of OTUs varied from 211 to 673 per sample (Supplementary Table S1).

**Figure 2:**
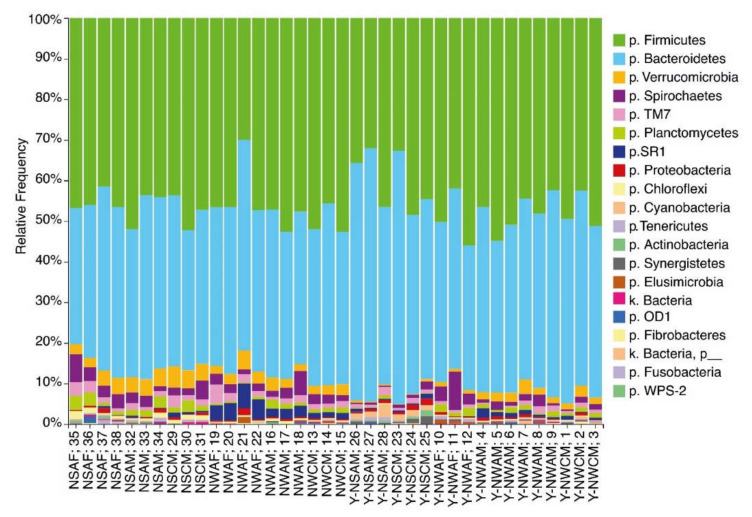
The taxonomic diversity (phylum level) of the ruminal microbial population in reindeer from Russian Arctic zones. Regions: N—Nenetski Autonomous District (AD); Y-N—Yamalo-Nenetski AD. Seasons: S—summer; W—winter. Age: A—adults; C—calves. Sex: M—male; F—female.

In all samples, the ruminal microbial population was dominated by two phyla, Bacteroidetes and Firmicutes, which represented 80.3% to 95.1% of the total microbial population (Figure 2). The percentage of Bacteroidetes varied from 33.6% to 62.4%; the average percentage of species from this phylum in animals from the NAD was significantly lower than that in animals from the YNAD (*p* = 0.05). The percentage of *Firmicutes* species varied from 30.0% to 56.0% without significant regional differences. These two phyla were followed by *Spirochaetes* (0.3–9.4%) and *Verrucomicrobia* (0.3–5.2%). The total percentage made up by the minor communities (*Tenericutes*, SR1, TM7, OD1, *Planctomycetes*, *Chloroflexi*, *Proteobacteria*, *Elusimicrobia*, *Actinobacteria*, *Fibrobacteres*, *Synergistetes*, *Fusobacteria*, *Cyanobacteria*) varied from 3.2% to 12.2%.

**Figure 3:**
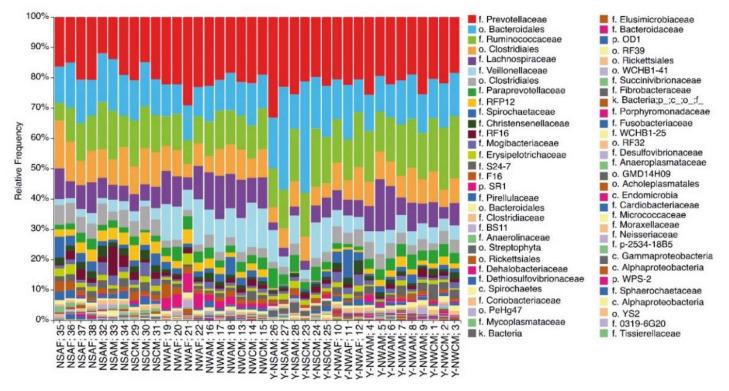
The taxonomic diversity (order level) of the ruminal microbial population in reindeer from Russian Arctic zones. Regions: N—Nenetski Autonomous District (AD); Y-N—Yamalo-NenetskiAD. Seasons: S—summer; W—winter. Age: A—adults; C—calves. Sex: M—male; F—female.

A more detailed taxonomic analysis (Figure 3) revealed the positions of dominance for the orders, as follows: Bacteroidales (33.6–62.4%) and Clostridiales (26.5–54.4%). The order Clostridiales was dominated by the families Ruminococcaceae, Lachnospiraceae, and Veillonellaceae; the order Bacteroidales was dominated by Prevotellaceae and Paraprevotellaceae. A significant trend for there to be a higher percentage of species from the families Lachnospiraceae and Dehalobacteriaceae (order Clostridiales) in the winter season in comparison with the summer season was found (*p* = 0.02).

### 3.2. The Alpha Biodiversity of the Ruminal Microbial Population in Reindeer

**Figure 4:**
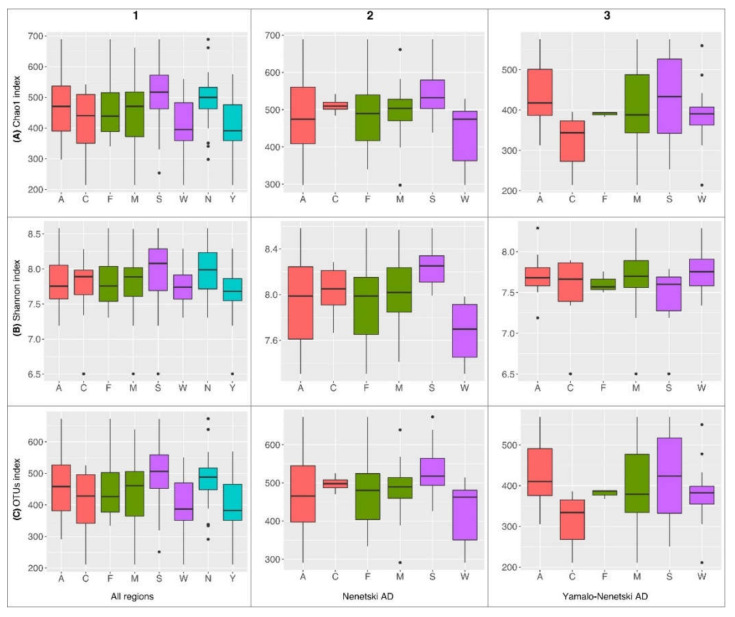
Box plot diagram of the alpha diversity indices of the ruminal microbial population in reindeer from Russian Arctic zones. Red plots reflect the differences between adults (A) and calves (C); green plots reflect differences between females (F) and males (M); purple plots reflect differences between seasons (S—summer, W—winter); and blue plots reflect differences between regions (N—Nenetski AD; Y—Yamalo-Nenetski AD). The first column of graphs (1) reflects the differences in both regions studied; the second column (2) reflects the internal differences in the Nenetski AD; the third column (3) reflects internal differences in the Yamalo-Nenetski AD. The first set of raw graphs (**A**) reflects the ChaoI index, the second (**B**) reflects the Shannon index, and the third (**C**) reflects the operational taxonomic unit (OTU) index.

The alpha diversity of the ruminal microbial population was characterized by Shannon’s (H) and Chao1 indices and the number of OTUs (Supplementary Table S2, Figure 4).

In samples from the YNAD, the Chao1 index was 403.01 ± 23.23, the H index was 7.64 ± 0.09, and there were 394.78 ± 22.98 OTUs. In samples from the NAD, the Chao1 index was 492.27 ± 21.82, the H index was 7.98 ± 0.08, and there were 479.45 ± 21.19 OTUs. All three indices differed significantly between regions: *p* = 0.004 for Chao1, *p* = 0.01 for H, and *p* = 0.005 for OTU.

In the summer samples, the Chao1 index was 503.31 ± 28.28, the H index was 7.94 ± 0.13, and there were 491.69 ± 27.48 OTUs; in the winter samples, the values were 411.21 ± 18.07, 7.73 ± 0.05, and 401.27 ± 17.59, respectively. Winter samples significantly differed from summer samples in terms of the Chao1 index (*p* = 0.01) and number of OTUs (*p* = 0.01). No significant differences in the indices of alpha diversity were found between samples from adults vs. calves and males vs. females.

The alpha diversity indices within each region followed different patterns. In the YNAD, the H index for winter samples (7.76 ± 0.07) significantly differed from summer samples (7.40 ± 0.20; *p* = 0.05). The number of OTUs and the Chao1 index differed significantly between calves and adults (314.50 ± 28.44 vs. 434.92 ± 24.51, *p* = 0.004, for the number of OTUs and 321.44 ± 29.55 vs. 443.79 ± 24.46, *p* = 0.004, for the Chao1 index).

In the NAD, only the seasonal differences between winter and summer were significant: 7.68 ± 0.09 vs. 8.26 ± 0.06, respectively, (*p* < 0.001) for the H index; 423.90 ± 25.03 vs. 535.00 ± 24.14 (*p* = 0.008) for the number of OTUs; and 435.71 ± 25.87 vs. 548.82 ± 25.09 (*p* = 0.008) for the Chao1 index. No significant differences were found in the NAD for calves vs. adults or for males vs. females.

The high H index values indicate a high level of the taxonomic diversity in the samples studied; the increases in Chao1 (characterizing the taxonomic diversity and higher shares of rare species) also indicate increases in the biodiversity of the ruminal microbial population.

### 3.3. The Beta Biodiversity of the Ruminal Microbial Population in Reindeer

**Figure 5:**
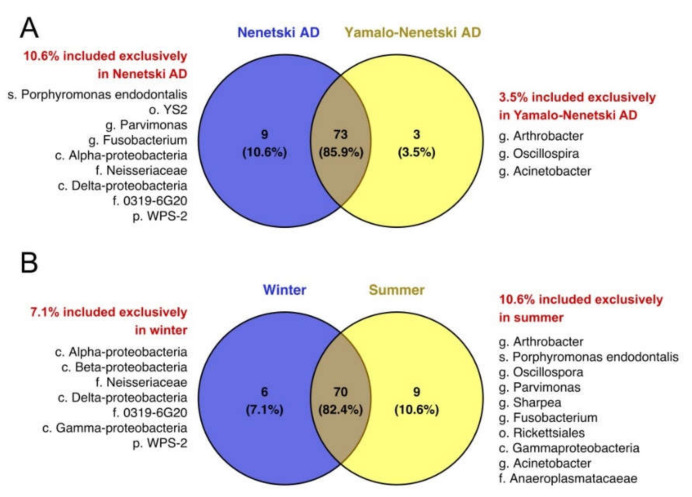
Venn diagram analysis of the diversity of the ruminal bacterial population in reindeer: comparisons of regions (**A**) and seasons (**B**).

The biodiversity of the ruminal microbial population was assessed by Venn diagram analysis (Figure 5). Some species were unique to certain regions studied (10.6% for NAD and 3.5% for YNAD); the percentage of common species was 85.9%. The percentage of unique species was influenced by season: it was higher in summer (10.6%) compared with winter (7.1%), while the percentage of common species was 82.4%. The species unique to the YNAD were *Porphyromonas endodontalis*, order YS2 of Cyanobacteria, *Parvimonas* spp., *Fusobacterium* spp., and certain Alpha-proteobacteria; for the NAD, unique species were *Arthrobacter* spp., *Oscillospira* spp., and *Acinetobacter* spp. Species unique to the summer season included *Arthrobacter* spp., *Oscillospira* spp., *Porphyromonas endodontalis*, *Parvimonas* spp., *Sharpea* spp., *Fusobacterium* spp., and *Acinetobacter* spp.; for winter, family 0319-6G20 of Myxococcales and certain Alpha-proteobacteria were unique.

**Figure 6:**
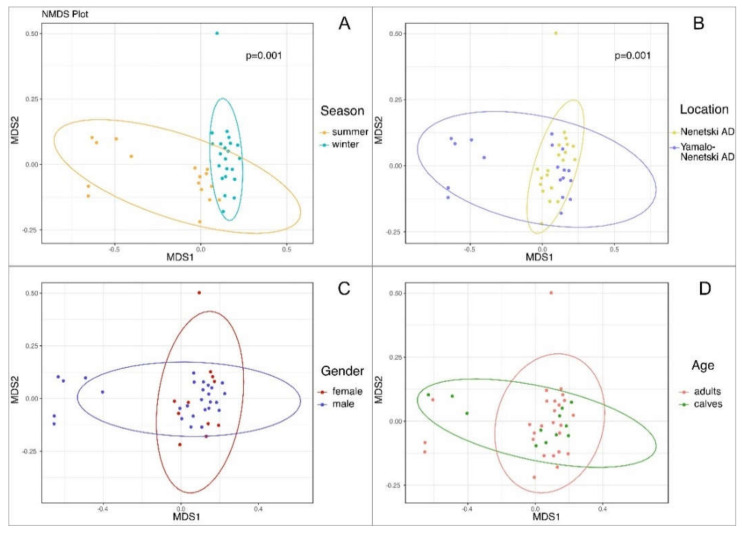
Non-metric multi-dimensional scaling (NMDS) of the samples from different seasons (**A**), regions (**B**), sexes (**C**), and ages (**D**).

The analysis of the beta diversity of the ruminal microbial population by NMDS (Figure 6) demonstrated evident clustering of the samples collected in different seasons. The substantial shift in summer vs. winter samples along the MDS1 axis confirms the uniqueness of the microbial population composition in different seasons. The seasonal differences were significant for all samples and for the samples from each separate region (*p* = 0.001). The comparison of samples from different regions also revealed significant clustering (*p* = 0.001).

### 3.4. The Effects of Ecological and Physiological Factors on the Ruminal Microbiota Composition

**Figure 7:**
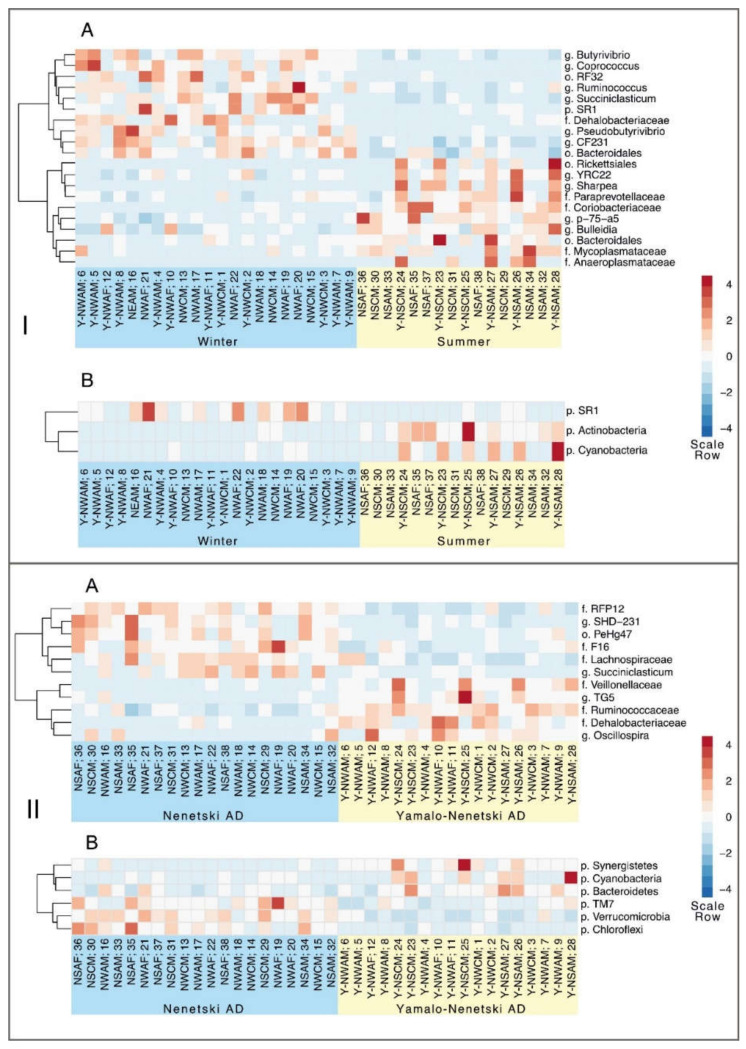
Heat map analysis of the taxa dominating the ruminal microbiome of reindeer from Russian Arctic zones in different seasons (**I**) and regions (**II**) at the levels of phylum (**A**) and genus (**B**). Regions: N—Nenetski Autonomous District (AD); Y-N—Yamalo-Nenetski AD. Seasons: S—summer; W—winter. Age: A—adults; C—calves. Sex: M—male; F—female.

The statistical analyses (alpha diversity indices, Venngraph, NMDS) indicated that region and season are the most influential factors significantly affecting the composition of the ruminal microbiota (Figure 4, Figure 5 and Figure 6). The effects of these two factors are summarized on the heat map at two levels, phylum and genus (Figure 7).

Season was found to significantly affect the presence of certain phyla. For example, in summer, the concentrations of Actinobacteria (0.35 ± 0.09 vs. 0.008 ± 0.006 in winter, *p* = 0.001) and Cyanobacteria (0.65 ± 0.22 vs. 0.05 ± 0.02 in winter, *p* = 0.02) were significantly higher than in winter, while the concentration of members from the SR1 phyla was significantly higher in winter (1.77 ± 0.36 vs. 0.40 ± 0.09 in summer, *p* = 0.02).

The analysis of regional differences at the phylum level indicated that in the NAD, the concentrations of members of the phyla Verucomicrobia (3.29 ± 0.24 vs. 1.3 ± 0.23 in the YNAD, *p* < 0.001), Chloroflexi (0.69 ± 0.1 vs. 0.23 ± 0.03 in the YNAD, *p* = 0.002), and TM7 (1.8 ± 0.22 vs. 0.88 ± 0.12 in the YNAD, *p* = 0.006) were significantly higher than in the YNAD, while in the YNAD, the concentrations of members of the phyla Cyanobacteria (0.58 ± 0.21 vs. 0.06 ± 0.02 in the NAD, *p* = 0.04), Synergistetes (0.34 ± 0.11 vs. 0.05 ± 0.02 in the NAD, *p* = 0.04), and Bacteroidetes (46.3 ± 1.81 vs. 40.91 ± 1.2 in the NAD, *p* = 0.05) were significantly higher than in the NAD.

The analysis of regional differences at the phylum level indicated that in the NAD, the concentrations of members of the phyla Verucomicrobia (3.29 ± 0.24 vs. 1.3 ± 0.23 in the YNAD, *p* = *p* < 0.001), Chloroflexi (0.69 ± 0.1 vs. 0.23 ± 0.03 in the YNAD, *p* = 0.002), and TM7 (1.8 ± 0.22 vs. 0.88 ± 0.12 in the YNAD, *p* = 0.006) were significantly higher than in the YNAD, while in the YNAD, the concentrations of members of the phyla Cyanobacteria (0.58 ± 0.21 vs. 0.06 ± 0.02 in the NAD, *p* = 0.04), Synergistetes (0.34 ± 0.11 vs. 0.05 ± 0.02 in the NAD, *p* = 0.04), and Bacteroidetes (46.3 ± 1.81 vs. 40.91 ± 1.2 in the NAD, *p* = 0.05) were significantly higher than in the NAD.

The analysis at the genus level revealed significant seasonal differences in bacteria from the family Coriobactriaceae (0.28 ± 0.05 in summer vs. 0.008 ± 0.006 in winter, *p* < 0.001). The concentration of members of the family Erysipelothrihaceae (1.0 ± 0.12 in summer vs. 0.5 ± 0.04 in winter, *p* < 0.001), including the genera *Sharpea* spp. (0.06 ± 0.02 in summer vs. 0 ± 0 in winter, *p* = 0.007) and *Bulleidia* spp. (0.34 ± 0.06 in summer vs. 0.13 ± 0.04 in winter, *p* = 0.02), and the family Mycoplasmataceae (0.20 ± 0.05 in summer vs. 0.06 ± 0.02 in winter, *p* = 0.03) were also significantly higher in summer. In winter, the concentrations of members of the family Dehalobacteriaceae (0.02 ± 0.01 in summer vs. 0.16 ± 0.04 in winter, *p* = 0.001), certain genera from the family Lachnospiraceae (*Butyrivibrio* spp. 0.86 ± 0.13 in summer vs. 3.18 ± 0.39 in winter, *p* < 0.001; *Coprococcus* spp. 0.14 ± 0.03 in summer vs. 1.07 ± 0.19 in winter, *p* = 0.003; and *Pseudobutyrivibrio* spp. 0.004 ± 0.003 in summer vs. 0.12 ± 0.03 in winter, *p* = 0.02), genus *Succiniclacticum* from the family Veillionelaceae (1.05 ± 0.24 in summer vs. 5.68 ± 0.85 in winter, *p* < 0.001), and genus *Ruminococcus* (0.27 ± 0.05 in summer vs. 0.77 ± 0.13 in winter, *p* = 0.02) were significantly higher than in summer.

The comparison of the regions at the genus level revealed that in the YNAD (wooded tundra), the concentrations of members from the families Ruminococcaceae (14.81 ± 0.59 vs. 10.34 ± 0.49 in the NAD, *p* < 0.001), Dehalobacteriaceae (0.19 ± 0.04 vs. 0.02 ± 0.01 in the NAD, *p* = 0.005), and Veillionelaceae (4.40 ± 0.82 vs. 1.37 ± 0.16 in the NAD, *p* = 0.007), genus *Oscilospira* spp. (0.38 ± 0.07 vs. 0.14 ± 0.05 in the NAD, *p* = 0.007) were significantly higher than in the NAD, while in the NAD (tundra) the concentrations of members of Verrucomicrobia (Verruco-5) (3.09 ± 0.23 vs. 1.22 ± 0.22 in the YNAD, *p* < 0.001), Anaerolinaceae (0.68 ± 0.10 vs. 0.23 ± 0.03 in the YNAD, *p* = 0.005), Planctomycetes from the order PeHg47 (0.23 ± 0.04 vs. 0.06 ± 0.02 in the YNAD, *p* = 0.005), Lachnospiraceae (4.3 ± 0.33 vs. 2.58 ± 0.35 in the YNAD, *p* = 0.009), and *Succiniclasticum* spp. (5.57 ± 0.98 vs. 1.69 ± 0.35 in the YNAD, *p* = 0.009) were significantly higher than in the NAD.

## 4. Discussion

Reindeer are rare animal species that can effectively survive on scarce plant food resources in the tundra and wooded tundra zones. The adaptive mechanisms (including the adaptation of the ruminal digestion) in reindeer allowing their inhabitation in areas of severe arctic conditions are currently under study [47,48,49,50]. We have presented data on the first array of the ruminal microbiota composition in reindeer from Russian Arctic zones (tundra of Yamalo-Nenetsky AD vs. wooded tundra of Nenetski AD) in relation to season, age, and sex using microbiome-wide NGS methodology.

The analysis of alpha indices revealed that the most influential factors affecting the composition of ruminal microbiota are region and season. All three indices significantly differed between regions, and two indices differed between seasons (Chao1 and the number of OTUs). The values of the alpha indices were higher in the NAD than in the YNAD and in summer samples compared with winter samples. High numeric values of these indices reflected greater taxonomic diversity in the ruminal microbial population, which acts as an evolutional adaptive mechanism to seasonal alterations in diets, allowing for prompt reaction to changes in food accessibility in different locations [12]. This adaptation is extremely favorable for survival in severe arctic conditions.

The taxonomic analysis of the ruminal microbiota provided evidence of the domination of the Bacteroidetes and Firmicutes phyla (from 80.3% to 95.1% of the total population; Figure 2). These phyla include the majority of anaerobes with different levels of activity in the degradation and fermentation of complex and simple carbohydrates from plant food ingredients. According to our present knowledge, these phyla dominate the ruminal communities of all ruminants, including reindeer. For example, the percentage of Bacteroidetes in reindeer has been reported to be the highest, accounting for above half of the ruminal population (61%), while the share of Firmicutes has been recorded as 30%. Minor communities include *Proteobacteria*, *Spirochaetes*, and *Chloroflexi* [51]. In the fecal microbial population in reindeer, [27] identified 14 phyla, and Firmicutes (56.53%) and Bacteroidetes (39.17%) were also found to dominate the population (~95% in total). The other 5% included members of the *Tenericutes*, *Cyanobacteria*, TM7, *Actinobacteria*, *Proteobacteria*, *Verrucomicrobia*, *Elusimicrobia*, *Planctomycetes*, *Fibrobacteres*, *Spirochaetes*, *Chloroflexi*, and *Deferribacteres phyla*. These data agree with our results, providing evidence of the dominant position of Bacteroidetes in the ruminal population, followed by Firmicutes.

Species from the phylum Cyanobacteria have been regularly identified and are minor (below 1%) components of the microbial populations in ruminants (cattle, camels) [52,53,54]. These species are common inhabitants of soil and water; therefore, their role in the rumen is still unclear. The presence of Cyanobacteria in the rumen could be related to the absorption of certain amounts of O2 and the fermentation of polysaccharides in the rumen under strictly aerobic conditions [55,56]. However, the percentage of these bacteria identified in our study was 3.2%; relatively high percentages of Cyanobacteria have also been found in other studies [27]. We can attribute the higher percentage of these bacteria in reindeer in comparison with other ruminants to the high level of lichens in the reindeer diet. According to Pankratov et al. [57], Cyanobacteria (primarily genus *Nostoc*, in lesser amounts, the genera *Calothrix*, *Scytonema*, and *Fischerella*) are typically symbiotic with lichens (cyanobionts) [58].

Our data show a significantly higher percentage of members of the phylum Cyanobacteria in the ruminal microbial population in reindeer from the YNAD in the summer season (1.1–3.2%) than in winter (0.5% or less). In the NAD, the percentage of these bacteria was 0.2% or less in summer, and the seasonal difference was insignificant. The higher ruminal percentage of Cyanobacteria in reindeer in comparison with other ruminants is apparently a unique feature related to the fact that the structural polysaccharides of lichens (lichenin, hemicellulose, xylan) considerably differ from the polysaccharides present in herbaceous and other higher plants [50,59]. The more severe tundra climate of the NAD results in a greater concentration of lichens in both winter and summer diets of reindeer, and therefore, the seasonal difference in the ruminal Cyanobacteria content in this region is less than in the YNAD, where the wooded tundra provides a more diverse diet.

Earlier, Henderson et al. [12] demonstrated that ruminal microbial populations in different ruminants have definite stable core community invariants for all studied ruminant species; this core community includes certain genera (*Prevotella*, *Butyrivibrio*, *Ruminococcus*) from the phyla Firmicutes and Bacteroidetes. Our data also provide evidence of the existence of a common ruminal community in reindeer, including members of the genera from the dominating phyla Firmicutes and Bacteroidetes. The percentages of other communities (Lachnospiraceae, Ruminococcaceae, Bacteroidales, Clostridiales), which can vary in relation to nutritional and/or environmental conditions, determines the specificity of different ruminant species [12], i.e., the interspecies differences indicating adaptation to different ecological niches are manifested at the level of minor taxa.

The NMDS beta biodiversity analysis of the ruminal microbiota in reindeer (Figure 6) revealed significant regional and seasonal clustering for all samples and for each region studied, indicating again that region and season are the most influential factors affecting the composition of the ruminal microbiota. These data agree well with the results of Mathiesen et al. [5], who demonstrated that the composition of ruminal microbiota differs between regional reindeer subspecies (mainland Norwegian vs. Svalbard) and seasons. These two factors are known to be the most influential for the ruminal microbial populations of different ruminant species [12]; however, for Arctic species, the seasonal effects can be more evident than regional ones, due to the significantly scarcer winter diets.

Different methods of data analysis (alpha diversity indices, NMDS, Venn diagram) convincingly indicated that region and season are the most influential factors affecting the composition of ruminal microbiota in reindeer at different taxonomic levels. Heat mapping (Figure 7) revealed a range of taxa that are susceptible to these two factors.

A significant seasonal difference in favor of summer was found for the family Coriobactriaceae. The concentration of species from this family in the gastrointestinal tract was reported to be related to stress [60]. The intestinal community of Coriobacteriaceae is known to transform the salts of bile acids and steroids and to activate dietary polyphenols; however, it can also be regarded as pathobiotic since it is related to certain pathologies, such as bacteremia, periodontitis, and vaginosis [61]. It is interesting to note that these data partially agree with the results of Zhou et al. [62] showing that ruminal *Coriobacteriaceae* is the taxon that is most susceptible to alterations in the composition of ruminal content. Significant seasonal differences in favor of summer were also found for the families Erysipelothrihaceae and Mycoplasmataceae. The Venn diagram analysis revealed certain taxa that were unique to the summer season, including pathogenic *Porphyromonas endodentalis* and *Fusobacterium* spp. The presence of pathogenic *Fusobacterium* spp. in the rumen of reindeer can induce certain diseases, including laminites. *Porphyromonas endodentalis* provokes the development of chronic periodontitis [63]. The higher concentrations of *Mycoplasmataceae* (involving a wide range of pathogens), *Porphyromonas endodentalis*, *Coriobacteriaceae*, and *Fusobacterium* spp. in the samples from the summer season could indicate that reindeer are more susceptible to different diseases in summer. Bach et al. [64] reported a positive correlation between the presence of members of *Erysipelothrihaceae* and the feed efficiency in cattle; the majority of *Erysipelothrihaceae* species, such as *Lactobacillus*, can apparently ferment a wide range of polysaccharides, with lactate being the dominating product [65].

The winter season was characterized by significantly higher concentrations of species from the family Paraprevotellaceae and genera *Butyrivibrio*, *Succiniclasticum*, *Coprococcus*, *Ruminococcus*, and *Pseudobutyrivibrio* than in summer. Recently the presence of *Paraprevotellaceae* in the rumen was reported as being correlated with the genetics of the host [66]. The authors attributed this correlation to the selective absorption of volatile (short-chain) fatty acids from the rumen for higher availability of energy for the host. *Coprococcus* species are butyrate-synthesizing and participate in other important metabolic processes in the rumen. A higher percentage of *Coprococcus* (*C. catus*) in the rumen was found in dairy cows with lower methane emissions and better feed efficiency. In cows with better feed efficiency, a higher abundance of lactate acrylate pathway genes for propionate transformation was found; in general, the acrylate pathway for propionate synthesis was found to be more effective than the succinate pathway, and it was dominant in the more productive cows [67]. Species from *Paraprevotellaceae* and *Coprococcus* are susceptible to external effects and are often regarded as indicatory [68,69]. Species from the genera *Butyrivibrio*, *Pseudobutyrivibrio*, and *Ruminococcus* are capable of activating the degradation of fiber roughage under anaerobic conditions; the enzymatic systems of the first two genera primarily produce butyrate, while the latter produces acetate and succinate [70]. Therefore, the increased amounts of these species in ruminal microbiota in winter are predictable and related to the increased share of roughage in the reindeer diet. Yamano et al. [71] also reported a significant winter-related increase in the concentration of *Ruminococcus flavefaciens* in the rumen of wild reindeer in Japan.

In the NAD (tundra zone), significantly higher concentrations of *Verrucomicrobia* (Verruco-5), *Anaerolinaceae*, *Planctomycetes* of order PeHg47, *Lachnospiraceae*, and *Succiniclasticum* spp. were found than in the YNAD. *Verrucomicrobia* and *Anaerolinaceae* are minor ruminal communities. Deusch et al. [72] reported the high metabolic activity of *Verrucomicrobia* in the rumen as being due to the higher protein content in comparison to other OTUs; the authors also underlined the minority of this ruminal community and the lack of knowledge regarding its role. It was also reported that the concentrations of these species in the rumen of Tibetan sheep tended to increase with age [73]. *Succiniclasticum* is a genus of ruminal bacteria that transforms succinate to propionate as the single energy-producing pathway; it also participates in the ruminal metabolism of fatty acids [74].

In the YNAD (wooded tundra zone), significantly higher concentrations of *Ruminococaceae*, *Dehalobacteriaceae*, *Veillionelaceae*, and *Oscilospira* spp. were found than in the NAD. The genus *Oscilospira* is still understudied; this genus of anaerobes belongs to the cluster Clostridia IV; however, there is a lack of knowledge regarding its metabolism and physiology. Recently, it was identified as a marker associated with certain interesting traits in humans, including leanness. This genus is supposedly butyrate-producing; certain species can utilize glucuronate, a common animal-derived saccharide that can be synthesized by humans and consumed in diets rich in animal-derived foodstuffs [75].

In the YNAD, a significantly lower concentration of members of the cellulolytic family Lachnospiraceae and significantly higher concentration of members from the cellulolytic family Ruminococaceae were found in comparison to the NAD. This probably indicates that reindeer from different regions consume different sources of cellulose depending on the climatic zone and specificity of the local vegetation. In other studies, diet has also been regarded as the basic factor affecting the composition of ruminal microbial population in ruminants [76].

The effects of age and sex on the composition of the ruminal microbial population in our study were found to be essentially insignificant. However, the analysis of alpha biodiversity revealed significant differences in the Chao1 and OTU indices between adult and young animals within the YNAD, indicating a higher degree of diversity in adults. This finding is in agreement with data from Jami et al. [77], who reported age-related increases in the alpha diversity indices and OTU number in the ruminal microbiota in cattle. The functionally matured rumen microbiota is, therefore, more taxonomically diverse than the rumens of juvenile animals.

Generally, significant differences in the composition of ruminal microbiota in reindeer were primarily related to regionally and seasonally dependent differences in dietary ingredients. Increases in the communities of certain pathogens (*Mycoplasmataceae*, *Fusobacterium* spp., *Porphyromonas endodentalis*) in summer were apparently related to abiotic factors (higher ambient temperatures and more active reproduction of parasites), while the increase in the community of cellulolytic species in winter was related to the increased contents of lichens and other types of roughage in the diet. Regional differences were primarily manifested as different ratios of cellulolytic species and species participating in the ruminal metabolism of volatile fatty acids; these ratios also reflect regional differences in the composition of vegetables in the reindeer diet.

## 5. Conclusions

The microbiome-wide array of the ruminal microbial population in reindeer from ecologically different Russian Arctic regions (wooded tundra of the YNAD and tundra of NAD) conducted in this study revealed region and season as being the most influential factors affecting the composition of the rumen. This is apparently related to the different diets consumed by reindeer in different regions and seasons. The diet of reindeer in the woodless tundra is primarily based on lichens and grasses, while in the wooded tundra, the diet is more diverse due to the larger amount of roughage from shrubs and trees. Seasonal changes in the microbiota due to the lower diversity of higher plants and domination of lichens in winter were also found to be significant. The summer-related increases in certain pathogenic communities in the rumen (*Mycoplasmataceae*, *Fusobacterium* spp., *Porphyromonas endodentalis*) reflect the higher susceptibility to diseases in this season. However, dietary change is evidently the most influential factor affecting the composition of the ruminal microbial population in reindeer from the Russian Arctic zone.

## Data Availability

Data are available in a publicly accessible repository.

## Supplementary Materials

The following are available online at https://www.mdpi.com/2076-2615/11/3/911/s1, Table S1: The characteristics of the samples from the reindeer, Table S2: The indices of alpha biodiverstable Sity of the ruminal microbial population between and within the regions.
