# Supplementary material for: The Comparative Analysis of the Ruminal Bacterial Population in Reindeer (Rangifer tarandus L.) from the Russian Arctic Zone: Regional and Seasonal Effects
Source: Animals (Basel). 2021 Mar 22;11(3):911. doi: 10.3390/ani11030911 (PMC8004722; doi:10.3390/ani11030911)
Supplement: Supplementary file 1 [file animals-11-00911-s001.pdf]

Table S1. The characteristics of the samples from the reindeers.

Sample name	Sequence count	OTU	Season	Region	Age	Sex
28SYAM	113156	531	Summer	YNAD	Adult	Male
27SYAM	97924	476	Summer	YNAD	Adult	Male
25SYCM	87740	319	Summer	YNAD	Calf	Male
32SNAM	86227	639	Summer	NAD	Adult	Male
37SNAF	84970	673	Summer	NAD	Adult	Female
33SNAM	81850	568	Summer	NAD	Adult	Male
31SNCM	77574	510	Summer	NAD	Calf	Male
30SNCM	76548	525	Summer	NAD	Calf	Male
38SNAF	57229	555	Summer	NAD	Adult	Female
35SNAF	56651	491	Summer	NAD	Adult	Female
29SNCM	54770	502	Summer	NAD	Calf	Male
26SYAM	53401	569	Summer	YNAD	Adult	Male
24SYCM	48463	371	Summer	YNAD	Calf	Male
34SNAM	44070	461	Summer	NAD	Adult	Male
23SYCM	41314	251	Summer	YNAD	Calf	Male
36SNAF	41268	426	Summer	NAD	Adult	Female
22WNAF	31759	514	Winter	NAD	Adult	Female
21WNAF	29221	334	Winter	NAD	Adult	Female
16WNAM	28141	388	Winter	NAD	Adult	Male
14WNCM	26760	485	Winter	NAD	Calf	Male
15WNCM	26538	494	Winter	NAD	Calf	Male
7WYAM	25829	550	Winter	YNAD	Adult	Male
22WNAM	25629	455	Winter	NAD	Adult	Male
14WNAF	24944	470	Winter	NAD	Adult	Female
11WYAF	22286	387	Winter	YNAD	Adult	Female
8WYAM	21080	478	Winter	YNAD	Adult	Male
17WNCM	20437	470	Winter	NAD	Calf	Male
12WYAF	19944	367	Winter	YNAD	Adult	Female
4WYAM	18571	433	Winter	YNAD	Adult	Male
13WNAF	17683	338	Winter	NAD	Adult	Female
10WYAF	16521	387	Winter	YNAD	Adult	Female
5WYAM	16308	357	Winter	YNAD	Adult	Male
21WNAM	14443	291	Winter	NAD	Adult	Male
3WYCM	13912	386	Winter	YNAD	Calf	Male
9WYAM	13109	379	Winter	YNAD	Adult	Male
6WYAM	12898	305	Winter	YNAD	Adult	Male
2WYCM	11420	349	Winter	YNAD	Calf	Male
1WYCM	5753	211	Winter	YNAD	Calf	Male

Table S2. The indices of alpha biodiversity of the ruminal microbial population between and within the regions.

**All regions**			**Yamalo-Nenetski Autonomous District (YNAD)**			**Nenetski Autonomous District (NAD)**		
**Index**		**p-value**	**Index**		**p-value**	**Index**		**p-value**
				**Shannon**				
YNAD	NAD							
7.64 ± 0.09	7.98 ± 0.08	0.0099965						
Winter	Summer		Winter	Summer		Winter	Summer	
7.73 ± 0.05	7.94 ± 0.13	0.2179046	7.76 ± 0.07	7.40 ± 0.20	0.0543353	7.68 ± 0.09	8.26 ± 0.06	0.0000523
Male	Female		Male	Female		Male	Female	
7.81 ± 0.08	7.84 ± 0.12	0.5228643	7.64 ± 0.11	7.61 ± 0.08	0.4226099	8.01 ± 0.10	7.93 ± 0.15	0.4976555
Calves	Adults		Calves	Adults		Calves	Adults	
7.76 ± 0.13	7.84 ± 0.07	0.5427709	7.49 ± 0.22	7.71 ± 0.08	0.3496629	8.03 ± 0.10	7.95 ± 0.11	0.7685305
				**OTU**				
YNAD	NAD							
394.78 ± 22.98	479.45 ± 21.19	0.0048891						
Winter	Summer		Winter	Summer		Winter	Summer	
401.27 ± 17.59	491.69 ± 27.48	0.0114793	382.42 ± 24.19	419.50 ± 51.26	0.3388186	423.90 ± 25.03	535.00 ± 24.14	0.0075542
Male	Female		Male	Female		Male	Female	
435.30 ± 20.33	449.27 ± 31.38	0.9991135	397.67 ± 27.65	380.33 ± 6.67	0.9586516	482.33 ± 24.90	475.13 ± 39.74	0.8484414
Calves	Adults		Calves	Adults		Calves	Adults	
406.08 ± 30.99	454.69 ± 19.76	0.0778701	314.50 ± 28.44	434.92 ± 24.51	0.0036984	497.67 ± 7.87	471.64 ± 30.19	0.5885066
				**Chao1**				
YNAD	NAD							
403.01 ± 23.23	492.27 ± 21.82	0.003875						
Winter	Summer		Winter	Summer		Winter	Summer	
411.21 ± 18.07	503.31 ± 28.28	0.0123744	390.78 ± 24.57	427.46 ± 51.65	0.3494124	435.71 ± 25.87	548.82 ± 25.09	0.0084612
Male	Female		Male	Female		Male	Female	
445.61 ± 20.90	460.72 ± 32.06	0.9997608	396.10 ± 27.98	389.95 ± 3.75	0.9835452	495.60 ± 25.85	487.26 ± 40.60	0.8311257
Calves	Adults		Calves	Adults		Calves	Adults	
416.30 ± 32.12	465.53 ± 20.21	0.081574	321.44 ± 29.55	443.79 ± 24.46	0.0036951	511.16 ± 8.07	484.17 ± 31.08	0.5963144
